# Anti-Endometriotic Effects of Pueraria Flower Extract in Human Endometriotic Cells and Mice

**DOI:** 10.3390/nu9030212

**Published:** 2017-02-28

**Authors:** Ji-Hyun Kim, Jeong-Hwa Woo, Hye Mi Kim, Myung Sook Oh, Dae Sik Jang, Jung-Hye Choi

**Affiliations:** 1 Department of Life and Nanopharamceutical Sciences, Kyung Hee University, 26 Kyungheedae-ro, Dongdaemoon-gu, Seoul 02447, Korea; erica32@hanmail.net (J.-H.K.); radiee@empas.com (J.-H.W.); msohok@khu.ac.kr (M.S.O.); dsjang@khu.ac.kr (D.S.J.); 2 College of Pharmacy, Kyung Hee University, 26 Kyungheedae-ro, Dongdaemoon-gu, Seoul 02447, Korea; hyemi586@gmail.com

**Keywords:** Pueraria flower, endometriosis, adhesion, migration, MMP

## Abstract

Pueraria flowers have been used as a vegetable and an ingredient for tea and jelly. In this study, we investigated the effects of Pueraria flower extract (PFE) on endometriosis, a common gynaecological disease characterised by local sterile inflammation of peritoneal cavity. PFE suppressed the adhesion of human endometriotic cells 11Z and 12Z to human mesothelial Met5A cells. In addition, PFE significantly inhibited the migration of 11Z and 12Z cells as shown by wound-healing and transwell migration assays. PFE reduced the protein and mRNA levels of matrix metalloproteinase (MMP)-2 and MMP-9 in endometriotic cells. Moreover, extracellular signal-regulated kinase (ERK)1/2 was activated by PFE treatment, and an ERK1/2 inhibitor, PD98059, significantly inhibited PFE-inhibited cell migration in endometriotic cells. Furthermore, PFE significantly suppressed endometriotic lesion formation in a mouse model. These data suggest that Pueraria flower is a potential anti-endometriotic agent for the inhibition of endometriotic cell adhesion, migration, and MMP expression.

## 1. Introduction

Endometriosis is a common gynaecological disease that affect approximately 1 in 7 women and 30%–50% of infertile women [1]. The common symptoms of the disorder are dysmenorrhoea, chronic pelvic pain, and infertility. Endometriosis is characterised by the ectopic implantation and growth of endometrial tissue and local sterile inflammation of peritoneal cavity. Proliferation, adhesion, and migration of ectopic endometrial tissue are required to establish endometriotic lesions in the peritoneal cavity [2,3]. In addition, the expression of matrix metalloproteinase (MMP)-2 and MMP-9 have been shown to be higher in women with endometriosis compared to healthy controls [4,5,6]. 

Treatment options for endometriosis include surgery and medication. Current medical treatments for endometriosis fall into pain medication such as non-steroidal anti-inflammatory agents or hormone therapy such as gonadotropin-releasing hormone (GnRH) agonists, and androgens. Although the treatments can improve some symptoms, there is no cure for endometriosis. The treatments do not reduce the high recurrence rate of endometriosis and can have several adverse effects such as genital atrophy and hot flashes [7]. Therefore, the establishment of novel and effective therapeutic agents is urgently needed to improve clinical management of endometriosis patients. 

*Pueraria lobata* Ohwi (*Leguminosae*), also known as “Kudzu” and “Japanese arrowroot” in the Western world, is a perennial leguminous vine endemic to eastern Asia [8]. In the United States, it is also called as “the vine that ate the South”, because the invasive vine covers 2.8 million ha across southeastern United States [9]. This plant produces an edible tuber, leaves, and flowers [8]. The Kudzu flowers have been used as a vegetable in Asian countries. The flowers tea is widely consumed in China. The flowers are also used as nutritional supplement for treatment of hangovers and obesity in Korea and Japan. In addition, the purple flowers of Kudzu are used an ingredient for sweet jelly in the southern United States. In Korean traditional medicine, Pueraria flowers are used to counteract symptoms associated with alcohol intoxication, liver injury, and menopause [10,11,12]. Modern pharmacological studies have shown that Pueraria flower has a wide range of biological activities, including hepatoprotective, antioxidant, hypoglycaemic, hypolipidaemic, antimutagenic, hormone-regulating, and anti-inflammatory effects [13,14,15,16,17,18]. However, there have been no scientific reports on the effect of Pueraria flower on endometriosis, a common gynaecological disease characterised by local sterile inflammation of the peritoneal cavity [19]. In this study, we investigated the effects of PFE on endometriosis as well as the associated molecular mechanisms.

## 2. Materials and Methods

### 2.1. Materials 

Flower of *Pueraria lobata* Ohwi was purchased from Inno Hanbang Herbal Drug Co. Ltd. (Seoul, Korea) and a voucher specimen of the herb (PF1-2012) was deposited in the herbarium of the College of Pharmacy, Kyung Hee University. Pueraria flower (100 g) was added to 10 times of distilled water, heat-extracted (for 2 h at 100 °C), and concentrated under reduced pressure. The filtrate was lyophilised using an FDU-550R freeze-dryer (Eyela Co., Tokyo, Japan), and stored at 4 °C until use. The dried extract of Pueraria flower (PFE) was dissolved in distilled water before each experiment. Foetal bovine serum (FBS), Dulbecco’s modified Eagle’s medium (DMEM)/F12 medium, penicillin G, and streptomycin were obtained from Life Technologies (Grand Island, NY, USA). CellTracker™ was purchased from Invitrogen (Grand Island, NY, USA). RNase, hydrocortisone, and PD98059 were obtained from Sigma Chemical Co. (St. Louis, MO, USA.). MMP-2 antibody was from Cell Signaling Technology, Inc. (Beverly, MA, USA). Antibodies for β-actin, MMP-9, total extracellular signal-regulated kinase (t-ERK), phospho-ERK (p-ERK), and the peroxidase-conjugated secondary antibodies were obtained from Santa Cruz Biotechnology Inc. (Santa Cruz, CA, USA). Enhanced chemiluminescence (ECL) system was purchased from Amersham Pharmacia Biotech (Oakville, ON, Canada).

### 2.2. Cell Culture and Cell Viability

Immortalised human endometriotic cells, 11Z and 12Z cells [20], were kindly provided by Dr. Starzinski-Powitz (Johann-Wolfgang-Goethe-Universitaet, Frankfurt, Germany). The endometriotic cells were cultured in DMEM/F12 medium supplemented with 100 U/mL penicillin G, 100 g/mL streptomycin, and 10% FBS. Met5A cells (human mesothelial cells) are originally from the American Type Culture Collection (Manassas, VA, USA). Met5A cells were cultured in Medium 199 medium supplemented with 100 U/mL penicillin G, 100 g/mL streptomycin, 10% FBS, and 400 nM hydrocortisone. The cells were maintained in a humidified atmosphere of 5% CO_2_–95% air at 37 °C. Cell viability was estimated using the MTT (3-(4,5-dimethylthiazol-2-yl)-2,5-dipheyl tetrazolium bromide; Sigma-Aldrich) assay. Cells were seeded in 96-well plates at a density of 5 × 10^3^ cells per well and incubated for 24 h. To examine the effect of PFE, cells were treated with PFE for 48 h. On the day of collection, 25 μL of MTT solution was added to the medium and the cells were incubated at 37 °C for 4 h. The MTT-containing medium was removed and the cells were solubilised in DMSO (100 μL) for 30 min. The absorbance at 490 nm was determined using a microplate spectrophotometer (Fisher Scientific Ltd., Ottawa, ON, Canada).

### 2.3. Adhesion Assay 

Met5A cells were grown to confluence on 96-well plates. Endometriotic cells (11Z and 12Z) were probed with CellTracker™ (10 mM) for 1 h at 37 °C and added (2 × 10^4^ cells/well) to the mesothelial Met5A cells on 96-well plates. Nonadherent cells were washed out with medium following incubation at 37 °C for 1 h. The fluorescence at a wavelength of 490 nm in each well was analysed. 

### 2.4. Wound-Healing Assay 

Scratch wound-healing assays were performed in 12-well tissue culture plates with minor modifications [21]. Endometriotic cells (11Z and 12Z) (5 × 10^5^ cells per well) were seeded in 12-well plates and grown to 80%–90% confluence. In order to create a denuded zone (gap) of constant width, the centres of the endometriotic cell monolayers were scraped with a sterile micropipette tip. After washing out the cellular debris with phosphate-buffered saline (PBS), cells were treated with PFE in the medium with 2% FBS. After 24 h treatment, the wound closure was photographed and analysed. The pictures of the initial wounded monolayers were compared with corresponding pictures of cells at the end of incubation. Artificial lines fitting the cut edges were drawn on pictures of the original wounds and overlaid on the pictures of cultures after incubation. Gap distance of the wound was measured using Photoshop software, and the data were normalised to the average of the control. Cells that migrated across the lines were counted in six random fields from each triplicate treatment, and data are presented as the mean ± SD.

### 2.5. Transwell-Migration Assay 

A 24-well transwell unit (8 μm pore size) with polyvinylpyrrolidone-free polycarbonate (PVPF) filters was used for the in vitro transwell-migration assay. Endometriotic cells (3 × 10^4^) were added in the upper part of the insert and incubated with the extract for 24 h at 37 °C. The cells that migrated to the lower surface of the membrane were stained with 0.05% crystal violet for 20 min after fixation with methanol. Migrative phenotypes were evaluated by counting the cells that migrated to the lower side of the filter under microscopy at 200×. All experiments were done in triplicate, and a minimum of five fields per filter was counted.

### 2.6. Western Blot 

After washing of the cells with ice-cold PBS, protein lysis buffer (0.5 mM Na orthovanadate, 5 mM EDTA, 0.1% Nonidet P-40, 250 mM NaCl, 0.5 mM dithiothreitol, 5 mM Na fluoride, 50 mM HEPES pH 7.0, 5 mg/mL leupeptin, 5 mg/mL aprotinin, 1 mM phenylmethylsulphonyl fluoride) was used to lyse cells. After mixing with 5× sodium dodecyl sulphate (SDS) sample buffer, the protein was boiled at 95 °C for 3 min, separated on 10%–12% SDS-polyacrylamide gels electrophoresis, and then electroblotted onto a polyvinylidene (PVDF) membranes. The membrane was immunoblotted using specific antibodies against β-actin, p-ERK, t-ERK, MMP-2, and MMP-9 in Tris-buffered saline containing 0.1% Tween-20 (TBS-T), followed by incubation for 1 h with a horseradish peroxidase-conjugated secondary antibody. The signals were visualised using ECL system and an Image Quant LAS-4000 (GE Healthcare Life Science, Wauwatosa, WI, USA).

### 2.7. Real Time RT-PCR 

Easy Blue^®^ kits (Intron Biotechnology, Seoul, South Korea) were used to isolate total RNA. Total RNA (0.5 μg) was reverse-transcribed (RT) using first-strand cDNA kit (Amersham Pharmacia Biotech, Oakville, ON, Canada). The primers used for SYBR Green real-time RT-PCR were as follows: for glyceraldehyde-3-phosphate dehydrogenase (GAPDH), 5′-GAGTCAACGGATTTGGTCGT-3′ and 5′-TTGATTTTGGAGGGATC TCG-3′; for MMP-2, 5′-ACCGCGACAAGAAGTATGGC-3′ and 5′-CCACTTGCGGTCA TCATCGT-3′; for MMP-9, 5′-CGATGACGAGTTGTGGTCCC-3′ and 5′-TCGTAGTTG GCCGTGGTACT-3′. Semiquantitative real-time PCR was carried out using Thermal Cycler Dice System (Takara, Otsu, Japan). A dissociation curve analysis of GAPDH, MMP-2, and MMP-9 showed a single peak. Mean cycle threshold (Ct) of the gene of interest was calculated from triplicate measurements and normalised with the mean Ct of a control gene, GAPDH. 

### 2.8. Animal Study

Female mice (5 weeks of age, BALB/c) were obtained from Korea Orient Bio, Inc. (Seoul, Korea). Animals were maintained with free access to food and water in pathogen-free conditions in an air-conditioned room (humidity of 55% ± 5% and temperature of 24 °C ± 1 °C) under controlled lighting (12 h light/dark cycle). All animal-handling protocols and surgical procedures were approved by the Committee for the Care and Use of Laboratory Animals (KHP-2014-05-1) in College of Pharmacy, Kyung Hee University in compliance with institutional guidelines for experimental animal care. The endometriosis was induced in mice using a previously established method with modifications [22,23]. Briefly, the uterine horns of the donor mice were removed and put into a dish containing PBS. The endometrium-rich fragments (1 cm) from the middle-third of the uterine horn were finely and uniformly chopped. The fragments (~20 pieces) suspended in PBS were injected into the peritoneal cavity of recipient mice with a micropipette to induce the formation of endometriosis-like lesions. The 15 mice with induced endometriosis were randomly divided into three groups (5 mice/group): control, PFE 150, and PFE 300. Mice were orally administered either vehicle (200 μL of PBS) alone or PFE (150 and 300 mg/kg/day) for 5 weeks from a week before the inoculation with endometrial fragments. Mice body weight changes were measured once per week during the treatment period. Additionally, we investigated whether the administration of PFE induced kidney and liver toxicity in the mouse models. After induction of endometriosis for 4 weeks, the mice were sacrificed by cervical dislocation and the peritoneum and visceral organs were examined visually to measure the number of endometriotic lesions (1 > mm). The serum creatinine and blood urea nitrogen (BUN) levels were measured as indicators of the kidney function, while the alanine transaminase (ALT) and aspartate transaminase (AST) levels were measured to evaluate the liver function. The levels of serum creatinine, BUN, and ALT/AST activity in the PFE-treated mice were not different from those observed in the vehicle-treated control mice.

### 2.9. Isolation of Major Compounds of PFE and UPLC Analysis

The water extract (37.56 g) was subjected to Diaion HP-20 column chromatography (CC) and eluted with a stepwise gradient of MeOH–H_2_O system (2:3 to 1:0, *v*/*v*) to afford 6 fractions (F1–F6). Compound 2 (247.2 mg) was purified from fraction F4 (4.86 g) by recrystallization in MeOH–H_2_O. The rest of fraction F4 was successively fractionated using a Sephadex LH-20 CC with MeOH–H_2_O mixture (4:1, *v*/*v*) and silica gel (230–400 mesh) CC with CH_2_Cl_2_–MeOH–H_2_O mixture (9:0.9:0.1 to 8:1.8:0.2, *v*/*v*/*v*) to yield compound 1 (149.4 mg). Fraction F6 (1.68 g) was subjected to a Sephadex LH-20 CC with MeOH–H_2_O mixture (4:1, *v*/*v*) to produce 10 subfractions (F6-1 to F6-10). Compound **3** (7.8 mg) was isolated from the fraction F6-8 by recrystallization in MeOH. The freeze-dried plant extract (1 mg) and each isolated compound (1 mg) were dissolved in DMSO and 50% MeOH solution (1 mL), and then filtered through 0.22 μm syringe filter. The mixture of standards (1 mg/mL) were diluted with 50% MeOH, produced various concentration (0.5 mg/mL, 0.25 mg/mL, 0.1 mg/mL, and 0.01 mg/mL). The UPLC Acquity system was conducted using a quaternary pump, auto-sampler, and photodiode array (PDA) detector with Waters Acquity UPLC Beh C18 (2.1 × 100 mm, i.d., 1.7 μm). Mobile phase consisted of water containing 0.1% formic acid (solvent A) and acetonitrile (solvent B) with gradient elution (linear gradient from 20% to 70% B in 7 min, followed by linear gradient from 70% to 100% B and isocratic for 1.5 min, and, finally, linear gradient from 100% to 20% B and isocratic elution for 3.5 min). The flow rate was 0.3 mL/min and the quantitative analysis was detected at 265 nm.

### 2.10. Statistical Analysis

For statistical analysis, GraphPad Prism version 5 (GraphPad, San Diego, CA, USA) was used. Statistical comparisons between two groups were made using Student’s *t*-test. For comparing more than two groups, one-way analysis of variance (ANOVA) was used followed by the Tukey test with a significance of *p* < 0.05. All errors are shown as standard deviation (SD).

## 3. Results 

### 3.1. PFE Treatment Inhibits Endometriotic Cell Adhesion to Mesothelial Cells

**Figure 1:**
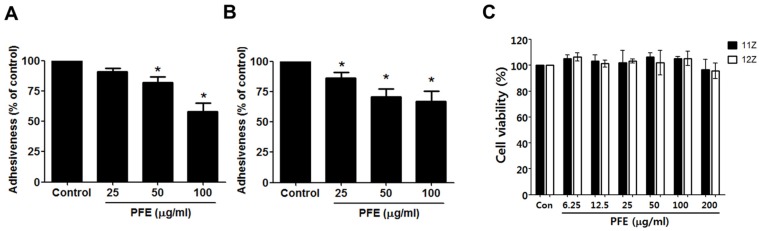
The effect of Pueraria flower extract (PFE) on endometriotic cell adhesion to mesothelial cells. Human endometriotic cells (11Z and 12Z) were labelled with CellTracker™ (10 mM). A confluent monolayer of Met5A cells was established on a 96-well plate. After 60 min of incubation of 11Z (**A**) and 12Z (**B**) cells at 37 °C with a Met5A cell layer, the total fluorescence in each well was measured by fluorescence microphotography. Cell adhesion was defined as the percentage of attached cells relative to control group cells. (**C**) Cell viability was determined by MTT assay. Data are presented as the means ± SD of three independent experiments. * *p* < 0.05.

The initial step of endometriotic lesion formation is the attachment of endometriotic cells to the layer of mesothelial cells that covers the peritoneal cavity. Using human endometriotic 11Z and 12Z cells and mesothelial Met5A cells, adhesion assay was performed to elucidate the effect of PFE on the adhesion ability of endometriotic cells to the mesothelial layer. Preincubation of 11Z and 12Z cells with PFE (25, 50, and 100 µg/mL) suppressed their adhesion to Met5A cells (Figure 1A,B). The antiadhesive effect of PFE was not attributable to any cytotoxic effect, since the extract, used at concentrations of up to 200 μg/mL, did not affect the viability of the endometriotic or mesothelial cells over a period of 48 h (Figure 1C).

### 3.2. PFE Inhibits Endometriotic Cell Migration

**Figure 2:**
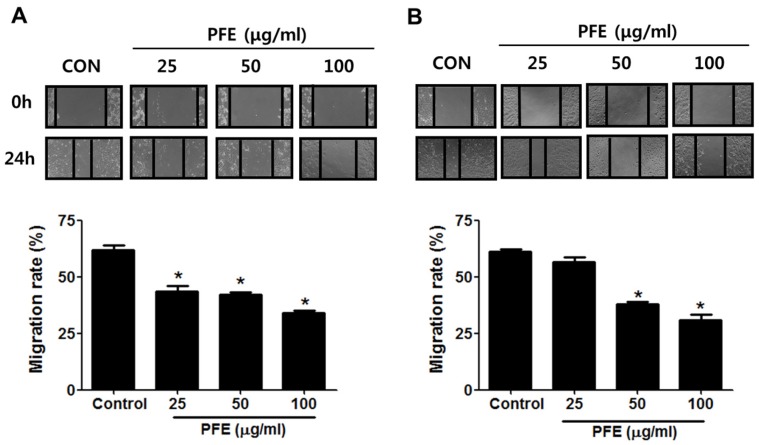
The effect of PFE on wound-healing ability in human endometriotic cells. Scratches were made in confluent cultures, and the endometriotic 11Z (**A**) and 12Z (**B**) cells were allowed to grow for 24 h following treatment with PFE (25, 50, 100 μg/mL). The distances covered by the cells (wound width) were plotted in terms of pixels. Data are presented as the means ± SD of three independent experiments. * *p* < 0.05.

**Figure 3:**
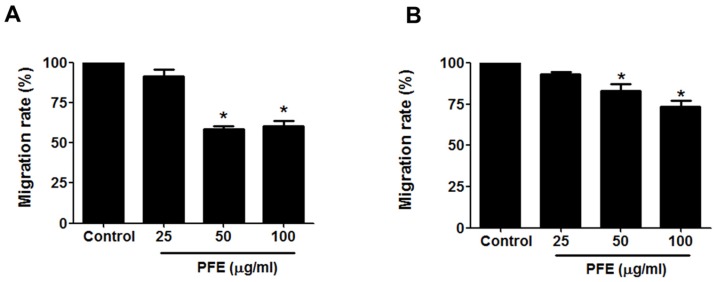
The effect of PFE on migration ability in human endometriotic cells. Human endometriotic 11Z (**A**) and 12Z (**B**) cells were seeded in uncoated chambers for migration assay in the presence or absence of PFE (25, 50, 100 μg/mL). Migrating cells were quantified using an inverted microscope as described in Materials and Methods. The columns represent the mean of three individual experiments performed in triplicate; bars, SD. * *p* < 0.05.

Wound-healing assays and transwell migration assays were performed to evaluate the effect of PFE on endometriotic cell migration. First, the wound-healing assay showed that the wound in endometriotic 11Z and 12Z cells was about 60% healed after 24 h in the absence of PFE treatment. In contrast, PFE significantly inhibited endometriotic cell migration compared with the control (Figure 2). Treatment of 11Z and 12Z cells with PFE at 100 µg/mL for 24 h resulted in only 30% migration. In addition, the migration assays demonstrated that PFE (25, 50, and 100 µg/mL) markedly suppressed the migration of human endometriotic cells (Figure 3). 

### 3.3. PFE Inhibits the Expression of MMP-2 and MMP-9 in Human Endometriotic Cells

**Figure 4:**
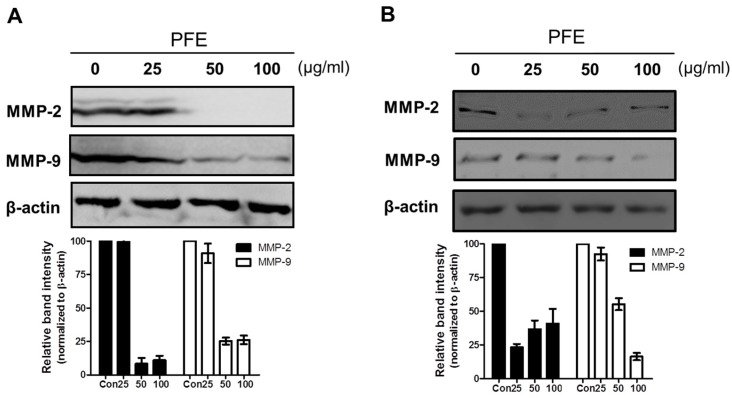
The effect of PFE on the protein levels of matrix metalloproteinase (MMP)-2 and MMP-9 in human endometriotic cells, Human endometriotic 11Z and 12Z cells were treated with PFE (25, 50, 100 μg/mL) for 24 h. Western blot assay was performed to measure the protein expression of MMP-2 and MMP-9 in 11Z (**A**) and 12Z (**B**) cells. β-Actin was used as a loading control. Data are shown as mean band density normalised relative to β-actin.

**Figure 5:**
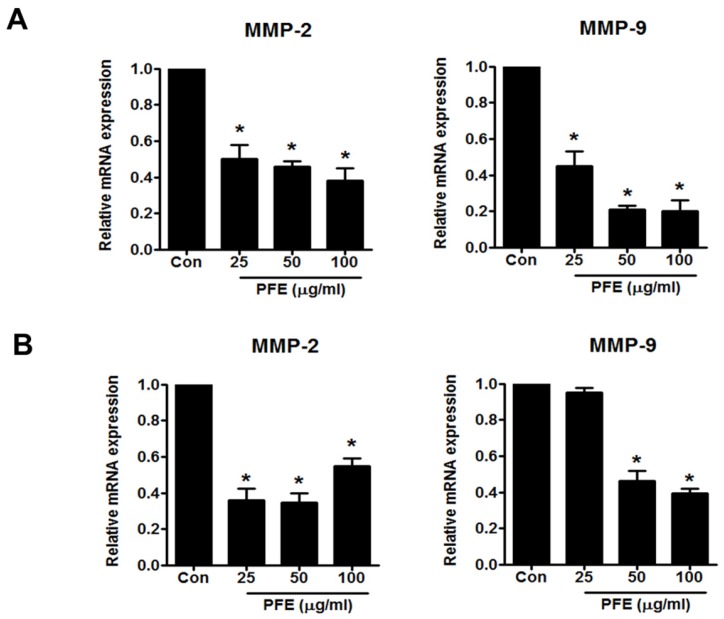
The effect of PFE on the mRNA levels of MMP-2 and MMP-9 in human endometriotic cells. Human endometriotic 11Z and 12Z cells were treated with PFE (25, 50, 100 μg/mL) for 24 h. Real-time RT-PCR was performed to measure the mRNA levels of MMP-2 and MMP-9 in 11Z (**A**) and 12Z (**B**) cells. Glyceraldehyde-3-phosphate dehydrogenase (GADPH) was used as an internal control. Data are presented as the means ± SD of three independent experiments. * *p* < 0.05.

MMP-2 and MMP-9 are well known to be involved in ectopic adhesion, invasion, implantation, and neovascularisation of the endometrium [24,25]. In this study, a Western blot assay was performed to measure the levels of MMP-2 and MMP-9 expression after 24 h of treatment with PFE (25, 50, and 100 μg/mL). PFE treatment markedly suppressed the levels of MMP-2 and MMP-9 protein in 11Z and 12Z cells (Figure 4). To investigate whether PFE regulates MMP-2 and MMP-9 expression at the transcriptional level, real-time RT-PCR was performed. As shown in Figure 5, PFE significantly inhibited the expression of MMP-2 and MMP-9 mRNA in human endometriotic 11Z and 12Z cells.

### 3.4. ERK Signalling Is Involved in PFE-Induced Anti-Endometriotic Activity

**Figure 6:**
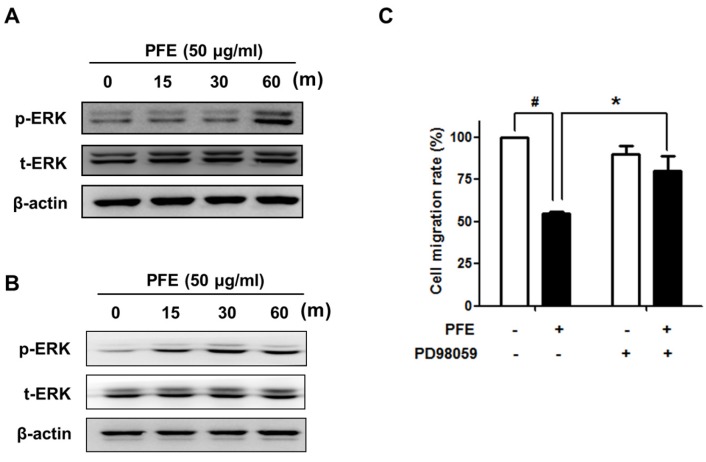
The effect of PFE on the phosphorylation of extracellular signal-regulated kinase (ERK)1/2 in endometriotic cells. 11Z (**A**) and 12Z (**B**) cells were treated with PFE (50 µg/mL) for an indicated time. Western blot analysis was performed to measure the levels of p-ERK1/2 and total (t)-ERK. (**C**) 12Z cells were pretreated with PD98059 (10 µM) for 30 min. After PFE treatment (50 µg/mL) for 24 h, transwell migration assay was performed. Data are presented as the means ± SD of three independent experiments. ^#^ *p* < 0.05 vs. the control; * *p* < 0.05 vs. PFE-treated group.

Several studies have demonstrated that the extracellular signal-regulated kinase (ERK) pathway is involved in cell migration and adhesion [26]. Western blot analysis revealed that PFE markedly increased the phosphorylation of ERK1/2 in endometriotic cells, as shown in Figure 6A,B. To investigate the involvement of ERK1/2 signalling in the PFE-inhibited cell migration of endometriotic cells, we performed a transwell migration assay in the presence of a specific ERK1/2 inhibitor, PD98059. As shown in Figure 6C, pre-treatment with PD98059 significantly reversed PFE-inhibited cell migration. These results indicate that, in endometriotic cells, PFE-inhibited migration may involve ERK1/2 signalling.

### 3.5. PFE Inhibits the Formation of Endometriosis-Like Lesions in Mice

**Figure 7:**
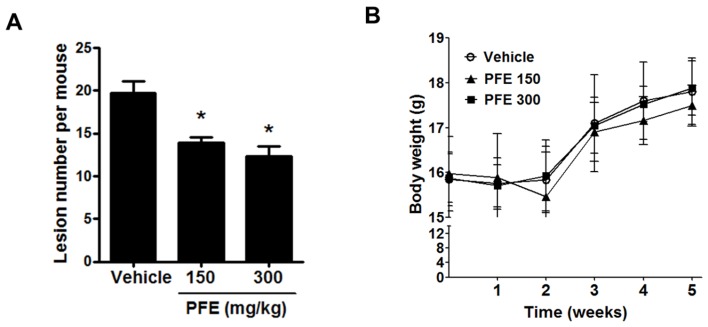
The effect of PFE on the formation of endometriosis-like lesions in mice. Mice were orally administered either vehicle (200 μL of phosphate-buffered saline (PBS)) alone or PFE (150 and 300 mg/kg/day) for 7 days before the inoculation with endometrial fragments. After induction of endometriosis for 4 weeks as described in Materials and Methods. (**A**) The numbers of endometriotic lesions were measured; (**B**) Mice body weight changes were measured once per week during the treatment period.

Four weeks after induction, mice were sacrificed to investigate the effect of PFE on the establishment of endometriosis-like lesions in vivo. As shown in Figure 7A, PFE-treated mice had a reduced number of total endometriotic lesions compared with vehicle-treated controls. Thus, PFE inhibits the formation of endometriosis-like lesions in mice. No significant weight loss or abnormal physiological change in mice treated with either vehicle or PFE was observed during the 5 weeks (Figure 7B).

### 3.6. Identification of Major Compounds of PFE and Anti-Endometriotic Effect of Tectorigenin

**Figure 8:**
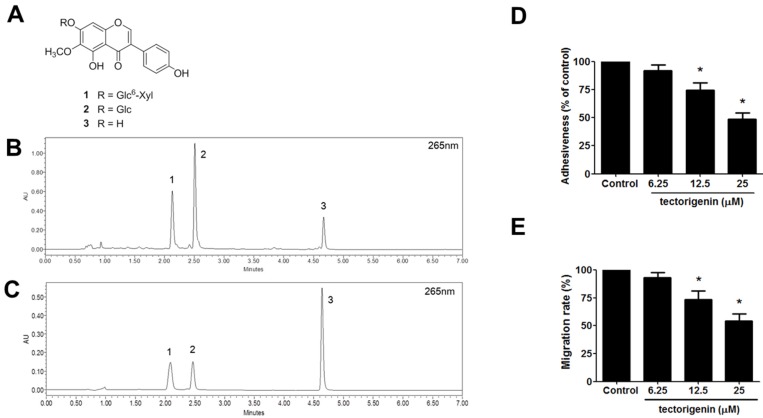
Identification of major compounds of PFE and anti-endometriotic effect of tectorigenin. (**A**) The structure of the three major isoflavones, tectorigenin-7-*O*-β-d-xylosylglucoside (**1**), tectoridin (**2**), and tectorigenin (**3**), isolated from PFE; (**B**) The UPLC chromatogram (at 265 nm) of PFE; (**C**) Reference standards of tectorigenin-7-*O*-β-d-xylosylglucoside (**1**), tectoridin (**2**), and tectorigenin (**3**); (**D**) Human endometriotic cells (12Z) were labelled with CellTracker™ (10 mM). A confluent monolayer of Met5A cells was established on a 96-well plate. After 60 min of incubation of 12Z cells at 37 °C with a Met5A cell layer, the total fluorescence in each well was measured by fluorescence microphotography. Cell adhesion was defined as the percentage of attached cells relative to control group cells; (**E**) Human endometriotic 12Z cells were seeded in uncoated chambers for migration assay in the presence or absence of tectorigenin. Migrating cells were quantified using an inverted microscope as described in Materials and Methods. The columns represent the mean of three individual experiments performed in triplicate; bars, SD. * *p* < 0.05.

Three major isoflavones **1**–**3** (Figure 8A) were isolated from the PFE by using repeated chromatography. These compounds were identified as tectorigenin-7-*O*-β-d-xylosylglucoside (**1**), tectoridin (**2**), and tectorigenin (**3**) on the basis of spectroscopic method (^1^H- and ^13^C-NMR) and by comparison with the published data. A mixed solution of three isoflavones and PFE extract were used for the optimisation of the UPLC condition. As shown Figure 8B,C, three isoflavones were separated within 7 min. Three isoflavones (peaks 1, 2, and 3) were identified by comparison of the retention time with those obtained from plant extract. The retention times of the three isoflavones 1–3 were 2.09 min, 2.47 min, and 4.64 min, respectively. Considering that tectorigenin-7-*O*-β-d-xylosylglucoside and tectoridin are generally metabolised into tectorigenin in the digestive tract, we investigated the effect of tectorigenin on the endometriotic cell adhesion and invasion. As shown in Figure 8D,E, tectorigenin significantly inhibited the cell adhesion and migration in endometriotic cells.

## 4. Discussion

Recent pharmacological studies have suggested that Pueraria flower has a wide range of biological activities, including hepatoprotective, antioxidant, hypoglycaemic, hypolipidaemic, antimutagenic, and hormone-regulating effects [14,15,16,17,18]. For example, Pueraria flower extract (PFE) was shown to have a protective effect on acute alcohol intoxication via modulation of alcohol metabolising and antioxidant enzymes in mice [27]. Liu et al. reported that PFE improves cognitive impairment in diabetic mice through the normalisation of acetylcholinesterase (AChE) activity and metabolic abnormalities in the brain [28]. Kamiya et al. demonstrated that PFE has anti-fatty liver and antiobesity effects in high-fat diet-induced obese mice by suppressing lipogenesis in the liver, stimulating lipolysis in white adipose tissue, and promoting thermogenesis in brown adipose tissue [29]. However, to the best of our knowledge, no report has demonstrated the effect of PFE on endometriosis. In this study, we first investigated the effect of PFE on endometriosis using human immortalised endometriotic 11Z and 12Z cells. The 11Z and 12Z cells were established from active endometriotic lesions and have been validated to maintain the phenotypic characteristics and in vivo properties of active phase endometriosis [20,30]. Endometriotic cells are characterised by enhanced proliferation, invasion, adhesion, and migration [30]. In this study, we demonstrated that PFE significantly inhibited the adhesion and migration of endometriotic cells with no significant change in cell viability. In contrast, any change in cell invasion was not observed following PFE treatment (data not shown), possibly due to the low invasiveness of 11Z and 12Z cells used in this study. Due to our promising in vitro results, the anti-endometriotic effect of PFE was examined in vivo using endometriosis-induced mice. PFE administration significantly disrupted the establishment of endometriosis-like lesions, suggesting that PFE should be considered as a potential preventive and therapeutic agent for endometriosis in humans.

MMPs, a family of zinc-dependent endopeptidases, are known to regulate the migration, invasion, and proliferation of various cell types [31]. Recently, accumulating data suggest that MMPs are associated with the establishment and progression of endometriosis. For example, MMP levels were shown to be enhanced in ectopic endometriotic tissues [32]. Styer et al. demonstrated that MMP levels in endometrial tissue are related to the ability of the tissue to progress into ectopic endometriotic lesions in a mouse endometriosis model [33]. Among the various MMPs, gelatinases MMP-2 and MMP-9, which degrade the principal component of basement membranes, collagen IV, have been intensively investigated in the context of endometriosis. MMP-2 and MMP-9 are elevated in the peritoneal fluid of patients with endometriosis compared to healthy people [5,34,35]. Similarly, induction of endometriosis enhanced the levels of MMP-2 and MMP-9 in peritoneal fluid and cells in an in vivo study using BALB/c mice [36]. In addition, human endometriotic tissues were shown to have higher levels and activities of MMP-2 and MMP-9 [4,37]. In this study, we showed that PFE significantly suppressed the mRNA and protein levels of MMP-2 and MMP-9 in both 11Z and 12Z cells, suggesting that PFE modulated MMP-2/-9 expression at the transcriptional level. Downregulation of MMP-2 and MMP-9 by PFE may involve the inhibition of PFE-induced migration in human endometriotic cells, considering that MMPs have been implicated in the endometriotic cell migration.

The mitogen-activated protein kinase (MAPK) pathways are well known to regulate a wide range of cellular events in eukaryotic cells. Activated MAPK coordinates diverse cellular activities including cellular metabolism, differentiation, cell cycle, motility, apoptosis, and survival. One of the MAPKs, ERK1/2, has been suggested as a key regulator of the majority of the signalling pathways and cascades associated with the pathogenesis of endometriosis [38]. For example, some factors in the endometriotic milieu such as tumour necrosis factor α (TNFα) and monocyte chemotactic protein-1 (MCP-1) have been shown to regulate ERK signalling in endometriotic cells [39]. In addition, Gentilini et al. have showed that ERK1/2 signalling pathway is associated with 17beta-estradiol and growth factor-induced cell migration of endometriotic stromal cells [40]. Interestingly, ERK1/2 has been implicated in the regulation of MMP expression [41,42,43]. In this study, we showed that treatment with PFE markedly increased the activation of ERK1/2 in endometriotic cells and that PFE-suppressed migration was significantly reversed in the presence of PD98059, a specific ERK1/2 inhibitor. The ERK1/2 activity remained elevated throughout the experiment (up to 24 h) (Supplementary Figure 1). These data suggest that ERK1/2 signalling is involved in PFE-inhibited endometriotic cell migration. 

Pueraria flowers are rich source of isoflavones, such as genistein, daidzein, kakkalide, puerarin, and tectoridin [44]. In fact, most of biological activities of the flowers have been attributed to the isoflavones. We found that tectoridin, tectorigenin-7-*O*-β-d-xylosylglucoside, and tectorigenin were the major components of PFE used in this study. This result is consistent with a previous finding that tectoridin is the major isoflavone component of Pueraria flowers during the first 5 years of storage [45]. Tectoridin and its major metabolite tectorigenin have been suggested to have a wide range of biological activities (e.g., antioxidant, anti-inflammatory, anti-angiogenic, anti-tumour, hepatoprotective, and endocrine-modulating effects) [12,46,47,48,49,50]. To the best of our knowledge, there have been no reports on the anti-endometriotic effects of the isoflavones of PFE. It is of note that tectorigenin-7-*O*-β-d-xylosylglucoside, and tectoridin are known to be metabolised into tectorigenin in the digestive tract [51], suggesting that the major isoflavones in the PFE are likely to be absorbed in to the bloodstream in the forms of tectorigenin. Like other isoflavones, tectorigenin, a type of *O*-methylated isoflavone, has been suggested to possess estrogenic activities [17]. Interestingly, tectorigenin has been shown to inhibit cell migration through downregulation of MMPs, including MMP-2 and MMP-9, in osteosarcoma cells [52]. In addition, tectorigenin has been demonstrated to exert some of their biological actions via the ERK1/2 pathway [53,54]. Additionally, we found that tectorigenin significantly inhibits the endometriotic adhesion and migration in this study. Thus, it is reasonable to speculate that the anti-endometriotic effects of PFE are mainly attributed to the major isoflavones. However, in vivo efficacy of the active constituents and its more exact molecular mechanisms of action still need to be further examined in future.

## 5. Conclusions

In summary, we demonstrate that PFE inhibits the adhesion and migration of endometriotic cells and the establishment of endometriosis-like lesions. The anti-endometriotic activity of PFE is thought to be associated with the downregulation of MMP-2 and MMP-9 and the regulation of ERK1/2 signalling. Taken together, these results suggest that PFE has potential adjuvant intervention for treating and preventing endometriosis.
